# Welding fume exposure is associated with inflammation: a global metabolomics profiling study

**DOI:** 10.1186/s12940-018-0412-z

**Published:** 2018-08-22

**Authors:** Sipeng Shen, Ruyang Zhang, Jinming Zhang, Yongyue Wei, Yichen Guo, Li Su, Feng Chen, David C. Christiani

**Affiliations:** 1 Department of Biostatistics, School of Public Health, Nanjing Medical University, Nanjing, 211166 Jiangsu China; 2 Department of Environmental Health, Harvard T.H. Chan School of Public Health, Boston, MA 02115 USA; 3 China International Cooperation Center for Environment and Human Health, School of Public Health, Nanjing Medical University, Nanjing, 211166 Jiangsu China; 4 Department of Medicine, Pulmonary and Critical Care Division, Massachusetts General Hospital and Harvard Medical School, 665 Huntington Avenue, Building I Room 1401, Boston, MA 02115 USA

**Keywords:** Boilermaker, Welding fume exposure, Inflammation, Metabolomics, Occupational health, Environmental exposures

## Abstract

**Background:**

Increasing evidence suggests that welding fume exposure is associated with systemic inflammation. Although celluar metabolites may be associated with inflammation, there is limited information on metabolomic changes during welding fume exposure. Such changes may play an important role in the occurrence, development, and prevention of metal-associated diseases. We aim to investigate human metabolomics changes pre- and post-welding fume exposure.

**Methods:**

This study included 52 boilermakers totally. We collected plasma samples pre- and post-shift welding fume exposure and prepared samples using the automated MicroLab STAR® system. Metabolite concentrations were measured using ultra performance liquid chromatography - tandem mass spectrometer (UPLC-MS/MS) methods. Two-way analysis of variance was used to test the significance of metabolite changes with false discovery rate correction.

**Results:**

Analysis detected several metabolic changes after welding fume exposure, mainly involved in the lipid pathway [glucocorticoid class (cortisol, corticosterone, and cortisone), acylcarnitine class, and DiHOME species (9,10-DiHOME and 12,13-DiHOME)], amino acid utilization (isoleucine, proline and phenylalanine), and S-(3-hydroxypropyl) mercapturic acid (3-HPMA). These compounds are all associated with inflammation according to previous studies. Further, additive interaction effects linked smoking and 3-HPMA levels. In the metabolite set enrichment analysis for diseases, the top two disease-associated metabolite pathways were systemic inflammation-related diseases including rheumatoid arthritis and systemic lupus erythematosus.

**Conclusions:**

This global metabolomics study shows evidence that metabolite changes during welding fume exposure are closely associated with systemic inflammation. The altered metabolites detected may be potential health monitoring biomarkers for boilermakers, especially for inflammation-related disease prevention.

**Electronic supplementary material:**

The online version of this article (10.1186/s12940-018-0412-z) contains supplementary material, which is available to authorized users.

## Background

Welding fumes comprise a wide range of non-metals and metals with varying toxic effects [1–3]. Epidemiological studies have demonstrated welding fume exposure is associated with various disease, including pulmonary disease, lung inflammation, cardiovascular disease, and cancer [4, 5]. An improved understanding of possible adverse health effects of exposure to welding fumes, as well as their underlying mechanisms, is important for risk assessment and the development of prevention strategies that will impact a large population of workers [6].

Metabolomics has been increasingly recognized as a powerful functional tool to understand complex biological machinery and to develop new biomarkers for environmental biomonitoring that can help prevent and treat environmental-associated diseases [7]. Metabolomics is based on comprehensive analysis of the endogenous low-molecular-weight biomolecules (typically < 1000 Da) [8] within a cell, tissue, or biofluid (e.g., plasma or urine) that are associated with different human metabolic processes [7]. Metabolomics applications are expanding in the field of occupational health as a fast and reproducible approach that directly reflects biological events related to exposure [9–12]. Therefore, monitoring disturbances of the metabolome is now more sensitive, easily accessible, less expensive, and more accurate [13].

Despite the availability of such applications, limited research has focused on systemic metabolomics alterations of welding fume exposure. Using the well-established occupational cohort of boilermaker construction workers, we interrogated biochemical profiles manifested in human plasma samples originating from boilermakers with occupational exposure to metal fumes, with the aim of characterizing metabolic migration from pre-exposure to post-exposure. We identified metabolite changes during welding fume exposure and further explored their potential biological functions in boilermakers.

## Methods

### Study design and data collection

We recruited 52 boilermakers at an apprentice welding school (Union Local 29, Quincy, MA) totally. All participants were selected from the well-characterized occupational cohort of boilermaker construction workers in eastern Massachusetts during 2010–2011, as previously described [12, 14]. Peripheral blood samples were collected from all subjects before (pre-) and immediately after (post-) a ~ 5-h welding workshop.

According to the welding time, blood sample were drawn in two batches to control the circadian variation [15]. Twenty-nine samples (batch 1) were collected in the morning (pre-) and afternoon (post-) while 23 samples (batch 2) were collected in the afternoon (pre-) and evening (post-) (Table 1). We provided the boilermakers with breakfast, lunch and dinner to control for nutrient intake as a confounder. Samples within each batch were collected in the same time over the same day. Same blood drawers and handling method were used for all samples.

**Table 1:** Demographic characteristics of the study population

Characteristic	Mean ± SD or N
Sample size	52^a^
Sample collecting time	
Morning and afternoon	29
Afternoon and evening	23
Age (years)	40.91 ± 12.22
Welding time (hours/month)	33.06 ± 25.51
BMI (kg/m^2^)	28.43 ± 5.41
Weight (kg)	89.53 ± 17.89
Height (m)	1.77 ± 0.08
Gender	
Male	50
Female	1
Race	
Caucasian	43
African American	4
Asian	2
Hispanic	2
Current smoker	
Yes	21
No	30
Medical history	
Diabetes	4
High blood pressure	5
Irregular heart arrhythmia	2
High cholesterol hyperlipidemia	7

^a^Baseline information of one sample was missing

### Sample accessioning

Following receipt, samples were inventoried and maintained at –80 °C. Each sample was accessioned into the Metabolon Laboratory Information Management System (LIMS) and was assigned by LIMS a unique identifier that was associated with the original source identifier only. This identifier was used to track all sample handling, tasks, results, etc. LIMS tracked all samples and derived aliquots. All portions of any sample were automatically assigned their own unique identifiers by LIMS when a new task was created, and the relationship of these samples was also tracked.

The purpose of Metabolon LIMS was to enable fully auditable laboratory automation through a secure, easy to use, and highly specialized system. The scope of Metabolon LIMS encompasses sample accessioning, sample preparation, instrumental analysis, reporting, and advanced data analysis. All subsequent software systems were grounded in LIMS data structures. It has been modified to leverage and interface with in-house information extraction and data visualization systems, as well as third-party instrumentation and data analysis software.

### Sample preparation

Samples were prepared using the automated MicroLab STAR system from Hamilton Company. Several recovery standards were added before the first step of the extraction process for QC purposes. To remove protein, dissociate small molecules bound to protein or trapped in the precipitated protein matrix, and to recover chemically diverse metabolites, proteins were precipitated with methanol under vigorous shaking for 2 min (Glen Mills GenoGrinder 2000) and then centrifuged. The resulting extract was divided into five fractions: two for analysis by two separate reverse-phase (RP)/ultra-performance liquid chromatography (UPLC)-MS/MS methods with positive ion-mode electrospray ionization (ESI); one for analysis by RP/UPLC-MS/MS with negative ion-mode ESI; one for analysis by HILIC/UPLC-MS/MS with negative ion-mode ESI; and one reserved for backup. Samples were placed briefly on a TurboVap (Zymark) to remove organic solvent. Sample extracts were stored overnight under nitrogen before preparation for analysis.

### Instrument variability control

Instrument variability was determined by calculating the median relative standard deviation (RSD) of internal standards that were added to each sample prior to injection into mass spectrometers (RSD = 4%). Overall process variability was determined by calculating median RSD for all endogenous metabolites (i.e., non-instrument standards) present in 100% of pooled matrix samples (RSD = 8%). RSD values met Metabolon’s acceptance criteria. More details are provided in the Additional file 1.

### Pathway enrichment analysis

To test the statistical significance of pathways, we performed a pathway enrichment analysis based on the hypergeometric test: $P\left(x=k\right)=\frac{\left(\frac{K}{k}\right)\left(\frac{N-K}{n-k}\right)}{\frac{N}{n}}$, where N is the total number of metabolites, n is the total number of significant metabolites, K is the number of metabolites in this pathway and k is number of significant metabolites in this pathway.

### Metabolite set enrichment analysis for disease

To identify and interpret patterns of human metabolite concentration changes with potential diseases, metabolite set enrichment analysis (MSEA) was used based on MetaboAnalyst [16]. We used the library of disease-associated metabolite sets in blood, which contained 416 metabolite sets reported in human blood.

*P*-values for pathway enrichment analysis and MSEA were adjusted by false discovery rate (FDR *q*-value) correction.

### Statistical analysis

In demographic descriptions, mean ± standard deviation (SD) or frequencies (n) were used to describe continuous variables or categorical variables. We followed these data preprocessing steps: normalization to volume extracted, imputation of missing values with the minimum observed value for each compound [17], and logarithmic transformation. Change of each metabolite during welding day was calculated as fold change value (FC): $FC=\frac{1}{n}\sum \limits_{i=1}^n\frac{\mathrm{post}\hbox{-} {\mathrm{exposure}}_i}{\mathrm{pre}\hbox{-} {\mathrm{exposure}}_i}$, where *i* represented different subjects. To test the difference of metabolites during exposure and take circadian variation into account, we used two-way analysis of variance (ANOVA) with interaction which included the welding exposure factor and circadian factor. In the results, we reported only the ANOVA results for the exposure effects.

Statistical analyses were performed using R version 3.3.0 (The R Foundation). *P* values were two-sided and FDR correction was calculated to consider multiple comparisons. *FDR-q* < 0.05 was considered statistically significant.

## Results

### Demographic descriptions of the study population

This study included paired samples (pre-exposure vs. post-exposure) of 52 boilermakers totally. In summary, they had an average age of 40.9 ± 12.2 years, ranging from 21 to 63 years; and an average BMI of 28.43 ± 5.41 kg/m^2^, ranging from 18 to 39 kg/m^2^. Only one individual was female, 42.0% were current smokers, and 83% were Caucasians (Table 1). No participants reported metal fever during the welding workshop.

### Metabolite alteration summary

**Fig. 1:**
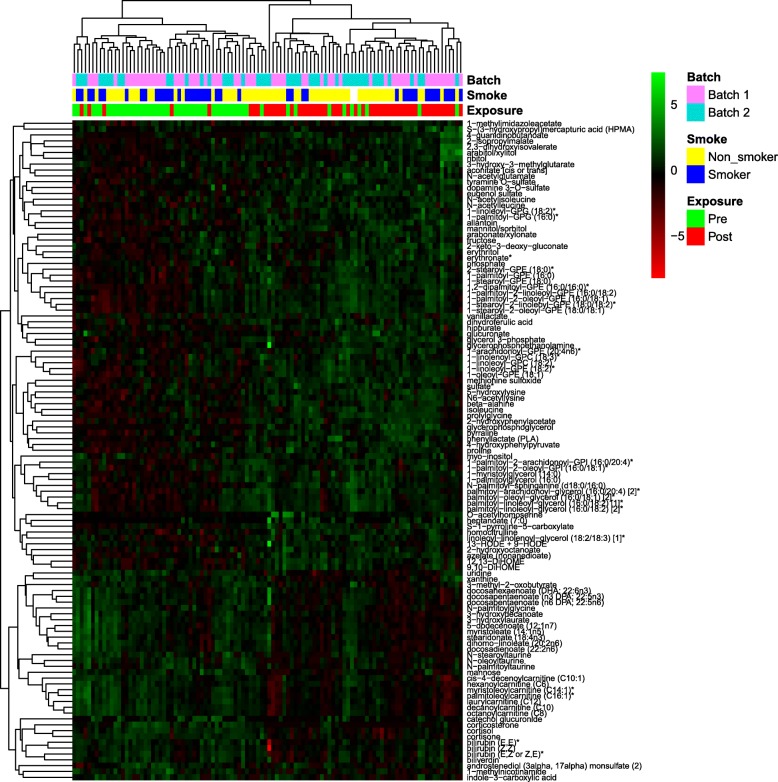
Heatmap of 113 significantly altered metabolites, hierarchically clustered for compounds (row) and samples (column). Batch information, smoking status, and exposure group are labeled at the top

We detected 693 known compounds in 8 metabolite superpathways and 77 subpathways (Additional file 2: Table S1). After quality control, imputation, normalization, and log transformation, we used two-way ANOVA to identify differences between pre-exposure and post-exposure groups. In summary, we identified 113 metabolites were significantly altered—78 were significantly up-regulated after exposure, while 35 were significantly down-regulated. Hierarchical clustering could distinguish between pre- and post-exposure workers (Fig. 1). For the circadian variation, 65 metabolites were significantly altered, including 41 up-regulated and 24 down-regulated compounds. However, no metabolites passed FDR correction for the interaction of exposure and circadian effects.

### Pathway enrichment analysis results

We identified 7 of 8 superpathways significantly enriched during the welding exposure, including lipid, amino acid, xenobiotics, carbohydrate, cofactors and vitamins, nucleotide, and energy pathways. For the subpathways, 14 of 41 pathways were significant with *q* < 0.05, such as lysolipid, phospholipid metabolism, fatty acid metabolism (acyl carnitine) and steroid pathways (Table 2).

**Table 2:** Significant superpathways and subpathways in pathway enrichment analysis

Pathway	Number	Different	Percentage (%)	Up^a^	Down^a^	P	FDR-*q*
Superpathway							
Lipid	321	61	19	37	24	2.18E-56	1.75E-55
Amino acid	158	22	13.92	19	3	7.37E-19	2.95E-18
Xenobiotics	97	10	8.77	10	0	9.41E-09	2.51E-08
Carbohydrate	21	8	38.09	7	1	4.04E-07	8.08E-07
Cofactors and vitamins	20	5	25	0	5	1.07E-04	1.71E-04
Nucleotide	30	4	13.33	2	2	6.76E-04	9.01E-04
Energy	9	2	22.22	2	0	2.64E-02	3.02E-02
Subpathway							
Lysolipid	24	10	41.67	10	0	1.91E-07	7.84E-06
Phospholipid Metabolism	33	8	24.24	8	0	4.47E-06	9.17E-05
Fatty Acid Metabolism (Acyl Carnitine)	23	7	30.43	0	7	2.14E-05	1.75E-04
Food Component/Plant	31	7	22.58	7	0	2.14E-05	1.75E-04
Phenylalanine and Tyrosine Metabolism	29	7	24.14	6	1	2.14E-05	1.75E-04
Polyunsaturated Fatty Acid (n3 and n6)	13	6	46.15	1	5	1.02E-04	6.94E-04
Diacylglycerol	19	5	26.32	5	0	4.79E-04	2.80E-03
Fatty Acid, Monohydroxy	14	4	28.57	2	2	2.24E-03	8.35E-03
Hemoglobin and Porphyrin Metabolism	5	4	80.00	0	4	2.24E-03	8.35E-03
Leucine, Isoleucine and Valine Metabolism	24	4	16.67	3	1	2.24E-03	8.35E-03
Steroid	36	4	11.11	0	4	2.24E-03	8.35E-03
Endocannabinoid	5	3	60.00	0	3	1.04E-02	3.05E-02
Fructose, Mannose and Galactose Metabolism	4	3	75.00	2	1	1.04E-02	3.05E-02
Pentose Metabolism	6	3	50.00	3	0	1.04E-02	3.05E-02

^a^Up indicates higher level in the post-exposure group; down indicates lower level in the post-exposure group

### Several classes of significant metabolite alterations

**Fig. 2:**
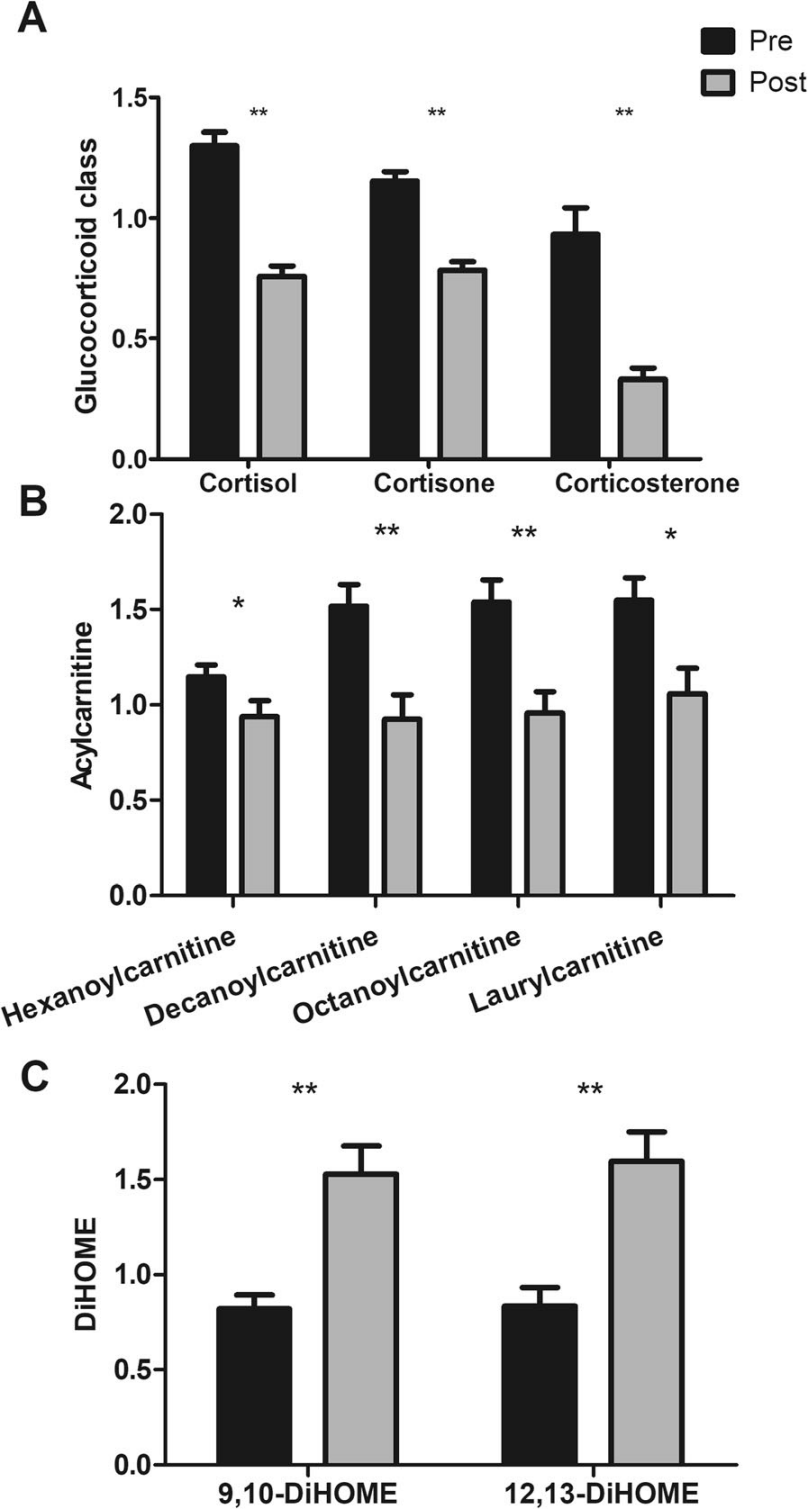
Pre- and post-welding fume exposure levels of (**a**) the glucocorticoid class of cortisol, cortisone, and corticosterone; (**b**) acylcarnitine species; and (**c**) DiHOME species 9,10-DiHOME and 12,13-DiHOME. Data are expressed as mean ± standard error of mean (SEM). **FDR *q*-value < 0.001

In the lipid pathway, post-exposure welders exhibited significant decreases in steroid hormones that were mainly involved in the glucocorticoid class [cortisol (FC = 0.58; *q* = 5.50 × 10^− 10^), cortisone (FC = 0.67; *q* = 2.43 × 10^− 8^), and corticosterone (FC = 0.35; *q* = 1.53 × 10^− 6^)] (Fig. 2a). In addition, the post-exposure group showed significant decreases in the acylcarnitine class [hexanoylcarnitine (FC = 0.81; *q* = 0.020), decanoylcarnitine (FC = 0.61; *q* = 8.75 × 10^− 5^), octanoylcarnitine (FC = 0.62; *q* = 3.49 × 10^− 4^), and laurylcarnitine (FC = 0.68; *q* = 0.001)] (Fig. 2b). Conversely, the post-exposure group had significantly higher levels of 9,10-DiHOME (FC = 1.87; *q* = 4.93 × 10^− 5^) and 12,13-DiHOME (FC = 1.91; *q* = 1.50 × 10^− 4^) (Fig. 2c).

In addition to the changes outlined above in the lipid pathway, the post-exposure group also exhibited a number of changes relating to complex lipid homeostasis. There were significant increases in lysolipids (10 of 24), phospholipids (8 of 33) and diacylglycerol (5 of 19) classes which may be related to increased utilization of the fatty acid pool.

Significant increases were observed in amino acid pathway profiles in the post-exposure group, including isoleucine (FC = 1.19, *q* = 0.008) and proline (FC = 1.18, *q* = 0.005). Phenylalanine also showed marginally significance (FC = 1.10, *q* = 0.079). Derivatives from these amino acids also significantly increased in the N-acetyl group, such as N-acetylglutamate, N-acetylleucine and N-acetylisoleucine, revealing high amino acid utilization.

**Fig. 3:**
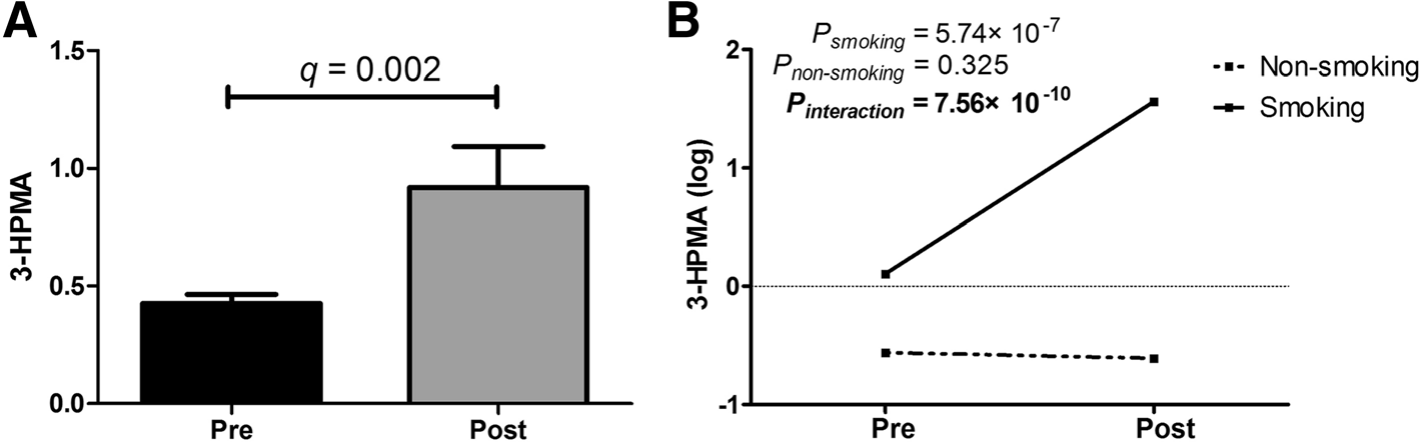
**a** Pre- and post-welding fume exposure levels of 3-HPMA. Data are expressed as mean ± SEM. **b** Interaction plot of 3-HPMA levels and smoking status for pre- and post-welding fume exposure groups. *P*_smoking_ indicates *t*-test *P*-value of 3-HPMA within the smoking subgroup, while *P*_non-smoking_ indicates *P*-value within the non-smoking subgroup. Two-way analysis of variance was used based on log-transformed data. *P*_interaction_ indicates *P*-value of interaction effects

Further, post-exposure samples had 2.16-fold increased levels of S-(3-hydroxypropyl) mercapturic acid (3-HPMA) (*q* = 0.032) (Fig. 3a). Considering that 3-HPMA could also be affected through tobacco smoke [18–20], we performed a stratification analysis by smoking status and found 3-HPMA was significantly increased in the smoking group (FC = 15.04; *P* = 5.74 × 10^− 7^) compared to the non-smoking group (FC = 1.09; *P* = 0.325) (Fig. 3b). Using two-way ANOVA for welding exposure and smoking status, additive interaction effects were also found between smoking and welding fume exposure (*P*_*interaction*_ = 7.56 × 10^− 10^).

To exclude the potential physiological confounding caused by sex differences, we performed a sensitivity analysis using only the male workers (*n* = 50). The significance of the altered metabolites remained (Additional file 2: Table S2).

### Metabolite set enrichment analysis (MSEA) for disease

**Fig. 4:**
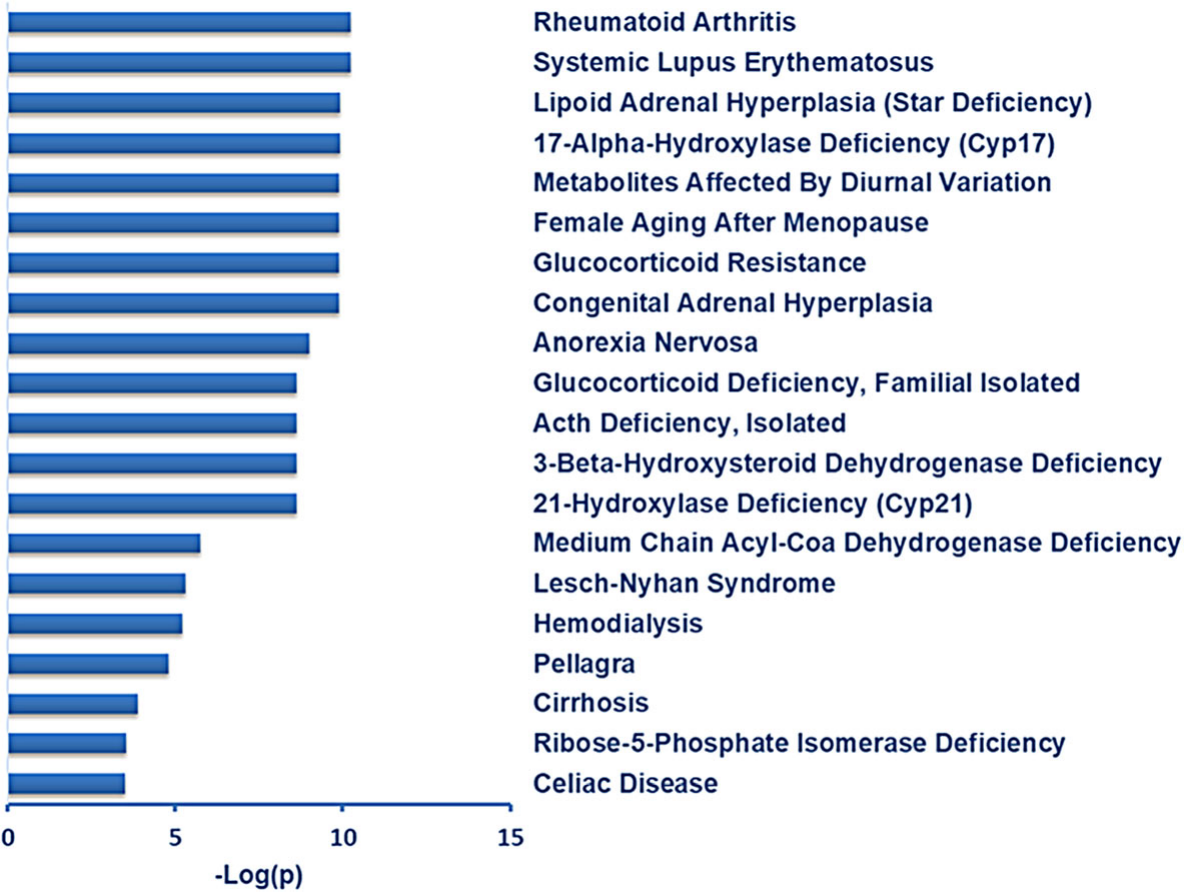
MetaboAnalyst metabolite set enrichment analysis of disease-associated metabolite sets in blood. Figure shows –log_10_(*p)* values for the top 20 significant pathways

Upon analysis of pre- and post-exposure compounds, MSEA for disease-associated pathways in blood showed 39 significant pathways, including rheumatoid arthritis (RA), systemic lupus erythematosus (SLE), and glucocorticoid resistance (Fig. 4, Additional file 2: Table S3).

## Discussion

Recently, studies have reported the relationship between welding fume exposure and inflammation. In this study, we investigated the global metabolomics profiling in human plasma for the first time. Our results revealed some different metabolite classes between exposure groups that were mainly in the lipid pathway (steroid hormones, acylcarnitine class, DiHOME species), amino acid utilization, and 3-HPMA. Interestingly, according to the previous studies [21–26]. These altered compounds showed a close relationship with inflammation, which may indicate an inflammatory mechanism of welding fume exposed boilermakers at the metabolomics level.

We observed significant decreases of glucocorticoid class including cortisol, cortisone and corticosterone as well as their hormone derivatives during welding fume exposure. Glucocorticoids, which belong to the steroid hormones family, are a class involved in regulating glucose metabolism, immunosuppressive, and anti-inflammatory responses in the body [26, 27]. Glucocorticoids are the most effective anti-inflammatory therapy for many chronic inflammatory and immune diseases [25]. Various evidence showed cortisol, cortisone and corticosterone played important roles in the inflammation [28–31]. Thus, monitoring the reductions of the glucocorticoid levels could provide evidence for relevant pulmonary and cardiovascular diseases.

We found significant decreases in the acylcarnitine class after exposure to welding fumes. The acylcarnitine class of metabolites often tracks with free fatty acids, as they are formed when fatty acids conjugate with carnitine. As important lipid biomarkers reflective of acyl-CoA status, acylcarnitines possess bioactive and inflammatory properties. Acylcarnitines have been reported as a marker of incomplete fatty acid β-oxidation and mitochondrial dysfunction [32]. In addition, alteration of acylcarnitines has an activating effect in many proinflammatory signaling pathways, and thus the compounds might have the potential to activate inflammation [24]. Acylcarnitines are also in insulin-resistance development, linking to muscle oxidative stress and inflammation [33]. Additionally, inflammatory bowel disease patients also have significantly lower acylcarnitine levels [34]. These results provide new evidence for an association with inflammatory diseases.

9,10-DiHOME and 12,13-DiHOME are derived from linoleate through epoxidation and hydration reactions and are thought to participate in mediating inflammatory responses [23]. They have been implicated in inflammatory disorders, such as adult respiratory distress syndrome [35], asthma [36], and may be endogenous regulators of vascular permeability and inflammation [37]. Their increased levels following welding fume exposure may provide new evidence for the association between DiHOME levels and systemic inflammatory responses [38].

Metabolic changes associated with inflammatory processes and immune responses can modify protein and amino acid requirements [39]. Such changes are usually considered the consequence of an increase in the production of cytokines that change protein metabolism. During the immunological stress process, amino acids are redistributed away from protein production towards tissues involved in inflammation and immune response [39]. We found that some groups of amino acids increased after exposure to welding fumes, which was consistent with a report that utilization of some amino acids increases during chronic inflammation [21].

3-HPMA is regarded as one of the main metabolites of acrolein, which is a toxic by-product of unsaturated aldehyde [40]. It is also a reliable biomarker to estimate acrolein concentration [41]. Acrolein exposure can occur through several mechanisms, including welding, particularly when the work involves painted materials and/or anticorrosive primers [42]. Main endogenous sources of acrolein are degradation of amino acids and polyamines, which may constitute a significant source of acrolein in situations of oxidative stress and inflammation [40]. Nevertheless, 3-HPMA levels are positively associated with cigarette smoking [19, 43]. We also found additive interactions between 3-HPMA and smoking that provide new evidence for further investigation of welding fume exposure and smoking-induced disease.

MSEA for disease linked metabolites with altered expression in the post exposure samples to different sets showed associations with various diseases. Of them, the top two disease-associated metabolite pathways were systemic inflammation-related diseases RA and SLE; the interaction was related to decreased cortisol and cortisone from steroid hormones. Several studies have reported that exposure to welding fumes containing metal is associated with systemic inflammation [44–46]. Cortisol and cortisone production are related to chronic inflammatory diseases, including RA and SLE [47], while welding fumes have also been reported as risk factors for RA [48, 49] and SLE [50]. Further, C-reactive protein (CRP) is considered a strong marker for systemic inflammation [51], and positive correlations are found within CRP and cortisol [52]. These results provide new evidence for an association between welding fumes and systemic inflammation at the metabolomics level.

We found several specific metabolites associated with inflammation, which may be potential health monitoring biomarkers for boilermakers, especially for inflammation-related diseases. Thus, health care products to boost immunity (e.g. vitamins) or anti-inflammatory could be useful in the boilermakers at risk to prevent the systemic inflammation and its consequences diseases.

However, we also recognize some limitations in the study. Firstly, though we considered circadian variation, this only reflected the differences between individuals at different collecting times, but not within individual variation. Secondly, we lacked important inflammation associated markers (e.g. CRP, white blood cell count, interleukin 6) to verify the relationship between metabolites and inflammation. Further studies are needed to validate the associations.

## Conclusions

In summary, this global metabolomics study shows the altered compounds including steroid hormones, acylcarnitine and DiHOME levels during welding fume exposure are associated with systemic inflammatory processes. The identified significant altered metabolites may be potential biomarkers for exposure-related inflammatory diseases among the boilermakers.

## Additional files

Additional file 1: Supplementary materials and methods. **Table S4.** Description of Metabolon QC samples. **Table S5.** Metabolon QC standards. **Table S6.** Data quality: Instrument and process variability. **Figure S1.** Preparation of technical replicates. (DOCX 120 kb)

Additional file 2: **Table S1.** Statistical summary of the 693 metabolites. **Table S2.**Sensitivity analysis that only keep the male workers. **Table S3.** MSEA analysis for disease-associated metabolite sets (blood). (XLSX 318 kb)

## Abbreviations

3-HPMA: S-(3-hydroxypropyl) mercapturic acid
ANOVA: Analysis of variance
CRP: C-reactive protein
FC: Fold change
FDR: False discovery rate
LIMS: Laboratory information management system
MSEA: Metabolite set enrichment analysis
RA: Rheumatoid arthritis
RSD: Relative standard deviation
SD: Standard deviation
SLE: Systemic lupus erythematosus
