# Supplementary material for: Welding fume exposure is associated with inflammation: a global metabolomics profiling study
Source: Environ Health. 2018 Aug 22;17:68. doi: 10.1186/s12940-018-0412-z (PMC6106842; doi:10.1186/s12940-018-0412-z)
Supplement: Supplementary file 1 — Supplementary materials and methods. Table S4. Description of Metabolon QC samples. Table S5. Metabolon QC standards. Table S6. Data quality: Instrument and process variability. Figure S1. Preparation of technical replicates. (DOCX 120 kb) [file 12940_2018_412_MOESM1_ESM.docx]

Additional file 1

## Materials and methods

**QA/QC:** Several types of controls were analyzed in concert with experimental samples: a pooled matrix sample generated by taking a small volume of each experimental sample (or, alternatively, a pool of well-characterized human plasma) served as a technical replicate throughout the data set; extracted water samples served as process blanks; and a cocktail of QC standards that were carefully chosen not to interfere with measurement of endogenous compounds were spiked into every analyzed sample, allowing monitoring of instrument performance and aiding chromatographic alignment (Tables S4 and S5). Instrument variability was determined by calculating median relative standard deviation (RSD) for standards that were added to each sample prior to injection into mass spectrometers. Overall process variability was determined by calculating median RSD for all endogenous metabolites (i.e., non-instrument standards) present in 100% of pooled matrix samples. Internal standards had a median RSD of 4%, and endogenous biochemicals had a median RSD of 8% (Table S6). Experimental samples were randomized across the platform run with QC samples spaced evenly among injections (Figure S1).

**Ultrahigh performance liquid chromatography-tandem mass spectroscopy (UPLC-MS/MS):** All methods utilized a Waters ACQUITY ultra-performance liquid chromatography (UPLC) and a Thermo Scientific Q-Exactive high resolution/accurate mass spectrometer interfaced with a heated electrospray ionization (HESI-II) source and Orbitrap mass analyzer operated at 35,000 mass resolution. Sample extract was dried and then reconstituted in solvents compatible to each of the four methods. Each reconstitution solvent contained a series of standards at fixed concentrations to ensure injection and chromatographic consistency. One aliquot was analyzed using acidic positive-ion conditions, chromatographically optimized for more hydrophilic compounds. In this method, extract was gradient-eluted from a C18 column (Waters UPLC BEH C18-2.1x100 mm, 1.7 µm) using water, methanol, 0.05% perfluoropentanoic acid (PFPA), and 0.1% formic acid (FA). Another aliquot was also analyzed using acidic positive-ion conditions; however, it was chromatographically optimized for more hydrophobic compounds. In this method, extract was gradient-eluted from the aforementioned C18 column using methanol, acetonitrile, water, 0.05% PFPA, and 0.01% FA and was operated at an overall higher organic content. Another aliquot was analyzed using basic negative-ion-optimized conditions with a separate dedicated C18 column. Basic extracts were gradient-eluted from the column using methanol and water, however with 6.5 mM ammonium bicarbonate at pH 8. The fourth aliquot was analyzed via negative ionization following elution from a HILIC column (Waters UPLC BEH Amide 2.1x150 mm, 1.7 µm) using a gradient consisting of water and acetonitrile with 10 mM ammonium formate at pH 10.8. MS analysis alternated between MS and data-dependent MS^n^ scans using dynamic exclusion. Scan range varied slighted between methods but covered 70–1000 m/z. Raw data files were archived and extracted as described below.

**Bioinformatics:** The informatics system consisted of four major components: the Laboratory Information Management System (LIMS), data extraction and peak-identification software, data processing tools for QC and compound identification, and a collection of information interpretation and visualization tools for use by data analysts. Hardware and software foundations for these informatics components were the LAN backbone and a database server running Oracle 10.2.0.1 Enterprise Edition.

**Data extraction and compound identification:** Raw data were extracted, peak-identified, and QC processed using Metabolon hardware and software. These systems are built on a web-service platform utilizing Microsoft’s .NET technologies, which run on high-performance application servers and fiber-channel storage arrays in clusters to provide active failover and load-balancing. Compounds were identified by comparison to library entries of purified standards or recurrent unknown entities. Metabolon maintains a library based on authenticated standards that contains retention time/index (RI), mass to charge ratio (*m/z)*, and chromatographic data (including MS/MS spectral data) on all molecules present in the library. Further, biochemical identifications were based on three criteria: retention index within a narrow RI window of the proposed identification, accurate mass match to the library +/- 10 ppm, and MS/MS forward and reverse scores between experimental data and authentic standards.

MS/MS scores were based on a comparison of ions present in the experimental spectrum to ions present in the library spectrum. While there may be similarities between these molecules based on one of these factors, use of all three data points can be used to distinguish and differentiate biochemicals. More than 3300 commercially available purified standard compounds have been acquired and registered into LIMS for analysis on all platforms for determination of their analytical characteristics. Additional mass spectral entries have been created for structurally unnamed biochemicals, which have been identified by virtue of their recurrent nature (both chromatographic and mass spectral). These compounds have the potential to be identified by future acquisition of a matching purified standard or by classical structural analysis.

**Curation:** A variety of curation procedures were carried out to ensure that a high-quality data set was available for statistical analysis and data interpretation. QC and curation processes were designed to ensure accurate and consistent identification of true chemical entities and to remove those representing system artifacts, mis-assignments, and background noise. Metabolon data analysts use proprietary visualization and interpretation software to confirm the consistency of peak identification among various samples. Library matches for each compound were checked for each sample and corrected if necessary.

**Metabolite quantification and data normalization:** Peaks were quantified using area-under-the-curve. For studies spanning multiple days, a data normalization step was performed to correct variation resulting from instrument inter-day tuning differences. Essentially, each compound was corrected in run–day blocks by registering the medians to equal 1.00 and normalizing each data point proportionately (termed “block correction”; Figure S2). For studies that did not require more than one day of analysis, no normalization was necessary, other than for purposes of data visualization. In certain instances, biochemical data may have been normalized to an additional factor (e.g., cell counts, total protein as determined by Bradford assay, osmolality, etc.) to account for differences in metabolite levels due to differences in amount of material present in each sample.

**Table S4.** **Description of Metabolon QC samples**

| **Type** | **Description** | **Purpose** |
| --- | --- | --- |
| MTRX | Large pool of human plasma maintained by Metabolon that has been extensively characterized | Assure that all aspects of the Metabolon process are operating within specifications |
| CMTRX | Pool created by taking a small aliquot from every customer sample | Assess effect of a non-plasma matrix on the Metabolon process and distinguish biological variability from process variability |
| PRCS | Aliquot of ultra-pure water | Process blank used to assess contribution to compound signals from the process |
| SOLV | Aliquot of solvents used in extraction | Solvent blank used to segregate contamination sources in the extraction |

**Table S5.** **Metabolon QC standards**

| **Type** | **Description** | **Purpose** |
| --- | --- | --- |
| RS | Recovery standard | Assess variability and verify performance of extraction and instrumentation |
| IS | Internal standard | Assess variability and performance of instrument |

**Table S6. Data quality: Instrument and process variability**

| ***QC sample*** | ***Measurement*** | ***Median RSD*** |
| --- | --- | --- |
| Internal standards | Instrument variability | 4% |
| Endogenous biochemicals | Total process variability | 8% |

**Figure S1.** Preparation of technical replicates. A small aliquot of each sample (colored cylinder) was pooled to create a CMTRX technical replicate sample (multi-colored cylinder), which was then injected periodically throughout the platform run. Variability among consistently detected biochemicals can be used to calculate an estimate of overall process and platform variability.

**Figure S2.** Visualization of data normalization steps for a multiday platform run.
